# Placebo Economics: A Systematic Review About the Economic Potential of Utilizing the Placebo Effect

**DOI:** 10.3389/fpsyt.2019.00653

**Published:** 2019-09-12

**Authors:** Jens Hamberger, Karin Meissner, Thilo Hinterberger, Thomas Loew, Katja Weimer

**Affiliations:** ^1^Department of Psychosomatic Medicine, University Clinic Regensburg, Regensburg, Germany; ^2^Division of Health Promotion, University of Applied Sciences Coburg, Coburg, Germany; ^3^Institute of Medical Psychology, Faculty of Medicine, LMU Munich, Munich, Germany; ^4^Department of Psychosomatic Medicine and Psychotherapy, Ulm University Medical Center, Ulm, Germany

**Keywords:** placebo effect, placebo response, cost-effectiveness, cost–benefit analysis, health economic evaluation

## Abstract

**Background:** Recent research shows that placebo mechanisms can be utilized in ethical and legal ways such as in open-label conditions, when patients know that they receive placebos, and through psychological interventions aiming to optimize patients’ expectations. Showing that placebo interventions are also cost-efficient could improve their acceptability.

**Objective:** To review studies that performed health economic evaluations (HEEs) of intentional placebo interventions and to review studies that intentionally applied placebo interventions and reported outcomes eligible for HEEs.

**Methods:** Two systematic reviews of the literature were performed. For the first review, we searched MEDLINE using “placebo” and Medical Subject Headings (MeSH) terms associated with HEEs such as “costs,” “cost–benefit analyses,” and “economics.” Studies were eligible if they employed patients, applied placebo interventions, included an appropriate control group, and reported results of cost analyses. For the second review, we searched the Journal of Interdisciplinary Placebo Studies (JIPS) database and MEDLINE using search terms for outcomes eligible for cost–utility analyses, such as “quality of life” or “quality-adjusted life years” (“QALYs”). Risk of bias of all studies found was assessed according to the *Cochrane Handbook*, and a narrative synthesis of the results is provided.

**Results:** The first search resulted in 1,853 articles, which were screened for eligibility. Two studies were found only in which costs or cost-effectiveness analysis were reported, but with medium to high risks of biases. The second search yielded 164 articles particularly from the JIPS database of which 11 studies met our search criteria: in six studies, patients received placebo pills in open-label conditions; three studies investigated effects of patient–physician relationships; and two studies used psychological interventions to optimize treatment expectations, in patients with various diseases and disorders. These studies report outcomes potentially eligible for HEEs when costs of interventions were known. Risks of biases were low to medium, but patients were not blinded to the conditions in most studies.

**Conclusions:** The state of knowledge about HEEs of placebo interventions is scarce. To gain more visibility and acceptability for placebo interventions, future studies should measure outcomes usable for HEEs and costs of interventions, and HEEs should be performed for existing studies if data are available.

## Introduction

During the last 20 years, placebo research investigated intensively the mechanisms by which placebo effects occur, but their utilization as a treatment option is still in its infancy (1, 2). One of the main reasons for this fact is—or was—that concerns about ethical and legal issues have been raised as the placebo use is often considered to involve deception of patients (3). Recent research, however, shows that placebo mechanisms can be used in ethical and legal ways such as in open-label conditions when patients know that they receive placebo pills (4, 5). Furthermore, a meta-analysis found similar effect sizes for placebos and active treatments (6). Showing that placebo interventions are not only effective but also efficient could further improve their visibility and acceptability, at least in certain circumstances, but little is known about health economic evaluations (HEEs) of placebo interventions (7). HEEs use various methods to analyze the efficiency of interventions either as total or relative costs or in relation to their effects.

Several studies could show that placebo interventions can improve symptoms of diseases by eliciting the underlying mechanisms such as influencing treatment expectations or learning of treatment effects through conditioning (1). In open-label placebo studies, patients are openly given placebos and are told that they can improve symptoms through self-healing mechanisms (4, 5). This has been shown, for example, for the treatment of irritable bowel syndrome (IBS) (8), low back pain (9), depression (10), and allergic rhinitis (11). In these studies, significant improvements of symptoms could be achieved while patients took no active drugs than in standard therapies, having the potential of reduced treatment costs. Studies using a so-called partial reinforcement schedule (1) showed that patients could be conditioned to drug effects and 50% drugs could be substituted for placebo pills while the effects of the full drug dose are maintained. This conditioning procedure has been shown to be effective for the substitution of stimulant drugs in attention deficit/hyperactivity disorder (ADHD) in children (12, 13) as well as for the substitution of corticosteroid therapy in psoriasis in adults (14). Furthermore, empathic practitioner–patient interactions have been shown to reduce the duration of the common cold by one whole day (15), which is a considerable economic factor. Although these studies comprised small sample sizes with fewer than 100 patients and short durations of maximal 3 weeks, they could show that placebo interventions can be applied successfully to patients. Additionally, a meta-analysis comparing differences between active treatment and placebo with differences between placebo and no treatment groups of three-armed trials found similar effect sizes for placebos and active treatments, particularly for continuous outcomes in 115 studies across different diseases (6). Despite such promising results, placebo interventions are far away from being considered as a treatment option, and HEEs of placebo interventions could support further research and acceptability (7).

HEEs are not part of approval procedures for new drugs but are more and more consulted for health-care decision making because of limited resources of health-care systems (16). To improve visibility and acceptability of placebo interventions, applying equal standards for testing their efficiency as for conventional drug therapy could be supportive. There are several methods for HEEs aiming to calculate health-care costs of an intervention in total or in relation to its effectiveness (16). The most frequently reported method is the cost–utility analysis (CUA), which measures the effects of an intervention with regard to its utility. To perform CUA, studies should assess the quality of life as outcome measure for the calculation of quality-adjusted life years (QALYs), that is, gained life years without symptoms. The cost-effectiveness analysis (CEA) utilizes clinical outcomes, morbidity, and mortality rather than quality of life measures and compares costs and effectiveness of an intervention with alternative interventions or placebo. For both CUA and CEA, an incremental cost-effectiveness or cost–utility ratio (ICER or ICUR, respectively) can be calculated as the ratio of additional costs divided by additional effectiveness of one intervention over another (ICER = (effect of intervention 1 − effect of intervention 0)/(cost of intervention 1 − cost of intervention 0)). Therefore, they provide information about extra costs per extra unit of the assessed effect or QALY. If intervention 1 is more effective than intervention 0, then a positive ICER indicates that intervention 1 is more expensive and a negative ICER indicates that intervention 1 is less expensive than intervention 0. For decisions in health care, thresholds have been proposed (but also criticized); for example, an ICER of up to £30.000 for a new drug or treatment is considered as cost-effective according to the National Institute for Health and Clinical Excellence (NICE) of Great Britain (17). Other methods are the cost-minimization analysis and the cost–cost analysis, which both compare costs of interventions when those are equally effective. An overview of different methods and their usage in different countries is presented by Riedel et al. (16). However, their overview shows that there is no established international standard for analyses or which outcomes should be reported in studies to perform HEEs.

To determine the current state about HEEs of placebo interventions, the primary aim of this article is to systematically review the evidence of HEEs of placebo interventions. As this review yielded only two studies, we additionally performed a second search to systematically review the literature to assess studies using placebo mechanisms that investigated outcomes that could at least be relevant for HEEs. Due to the lack of standard methods for economic evaluations and as we aim to provide a comprehensive review of the literature, a broadly based literature research was performed.

## Materials and Methods

This systematic review was performed in accordance with the Preferred Reporting Items for Systematic Reviews and Meta-Analyses (PRISMA) statement (18, 19) (**Supplement 1**), except a previous registration of the research protocol.

### Review Process

All literature researches were performed with regard to previously defined search criteria by two independent reviewers (JH and KW). In case of different search results, they were compared and discussed to come to an agreement. Lists of found articles were transferred from MEDLINE/PubMed to the reference management software EndNote™ (Version X7; Thomson Reuters), and duplicate articles, articles published in any other language than English or German, and letters, editorials, and comments were excluded. We restricted our search to articles published in and after 1995, because the term “placebo effect” [except in randomized controlled trial (RCTs)] as well as the systematic investigation of its underlying mechanisms was seldom reported before the mid-1990s (20), and current methods of HEEs are even younger. Of all remaining articles, titles were screened for eligibility. If the title did not suffice for a decision, abstracts were screened. Literature researches were performed between October and November 2018 and updated before submission on March 8, 2019.

### Search and Eligibility Criteria

To answer the first question, whether and with which results HEEs of placebo interventions have been performed and reported, MEDLINE/PubMed was screened for “placebo” in addition to search terms suggested by Droste and Dintsios (21). They provided a list of Medical Subject Headings (MeSH) terms related to HEEs of which 53 relevant MeSH terms were selected for our systematic review (**Supplement 2**). Due to the large number of search terms, each search was performed separately, and double entries were excluded in a second step. The following search term was finally used with “xxx” as a placeholder for MeSH terms listed in **Supplement 2**: (“placebos”[MeSH Terms] OR “placebos”[All Fields] OR “placebo”[All Fields]) AND “xxx”[MeSH Terms].

Titles, and abstracts if necessary, were screened for any evidence about HEEs of placebo effects or placebo responses as the topic of the article, whether in RCTs or placebo studies. As we aimed to reach and provide a broad overview about HEEs of placebo interventions, we predefined only a few eligibility criteria according to the PICOS (population, intervention, comparator, outcomes, study design) approach (18, 19). Studies were considered if they employed patients with any disease or disorder, but studies with healthy volunteers were excluded. Interventions were considered if they aimed to improve any disease or disorder by means of an intentional placebo intervention that was explicitly stated as such by the article’s authors or was recognized as such by the reviewers (JH and KW). An appropriate comparator group for placebo effects, such as a no-treatment or waiting list group, must have been included. Results of a HEE must have been reported in the article, for example, total or incremental costs of interventions, ICER or ICUR, or QALY. All kinds of study designs were considered such as randomized and non-randomized clinical trials.

For the second question, whether there are studies investigating placebo mechanisms reporting outcomes suitable for HEEs, the Journal of Interdisciplinary Placebo Studies database (JIPS; https://jips.online) (20) was screened first. This database was founded by Enck and colleagues and contains 4,174 articles (on February 28, 2019) dealing with the placebo effect and related topics only. Articles included are hand-selected by Paul Enck and Katja Weimer from PubMed on a weekly basis; for a detailed description of the selection process, see Enck et al. (20). Eligibility criteria according to the PICOS approach (18, 19) were as follows: studies involving patients with any disease or disorder (Population), with a planned and intentional placebo intervention (Intervention) compared with an appropriate control group for unspecific effects such as regression to mean (Comparator), assessing outcome parameters allowing for HEEs (Outcomes), and in which patients were randomized to the interventions (Study design). According to Riedel et al. (16), outcome parameters of studies eligible for HEEs are not well defined. However, quality of life is considered the most important outcome parameter as well as morbidity and mortality. We therefore searched for “quality of life,” “QoL,” “disability,” and common measures of this entity such as “SF-36” (“SF36”), “SF-12” (“SF12”), and “EQ-5D” (“EQ5D”) and for “morbidity,” “mortality.” Additionally, we searched for “quality-adjusted life years” (“QALY”) and “disability-adjusted life years” (“DALY”). The JIPS database was used for the second question, as the first systematic review reported above yielded a great amount of search results with the search term “placebo” but with low specificity for intentional placebo interventions, and a second literature search with this term was considered inefficient. However, to confirm this search, MEDLINE/PubMed was screened for each “placebo effect,” “placebo response,” and “placebo treatment” in combination with all of the above-mentioned search terms for outcome parameters (see **Supplement 3** for the full search term) and were searched for the above-described PICOS criteria.

### Data Extraction

The following data of eligible articles were extracted (**Tables 1** and **3**): condition (disease or disorder), applied intervention, control group used, number of patients involved, age and sex of patients, outcome measures, and results (results in **Table 1** only).

**Table 1:** Studies reporting health economic evaluations for placebo interventions.

Study	Condition	Placebo intervention	Control group	No. of patients (male:female)	Age (years; M ± SD)	Outcome measure for HEEs	Results
Gupta et al. (23)	Anxiety behavior and compliance while anesthesia	Anesthetic mask with flavor of patients’ choice	Anesthetic mask without flavor	60 (45:15)	7.1 ± 2.3	Direct and indirect costs for flavored masks	Anxiety and compliance did not differ; higher overall costs for flavored masks compared with non-flavored
Pattamatta et al. (24)	Postoperative ileus and anastomotic leakage after colorectal surgery	Gum chewing	Placebo dermal patch	120 (84:36)	66.5 ± 10.0	Total costs, costs for ward stay, ICER	Positive ICER in favor of gum chewing (lesser costs and positive effects)

HEEs, health economic evaluations; ICER, incremental cost-effectiveness ratio.

### Quality Assessment

Risk of bias of identified studies was assessed in accordance with the *Cochrane Handbook for Systematic Reviews of Interventions* (22) with regard to the following quality features of studies: random sequence generation (selection bias), allocation of concealment (selection bias), blinding of participants and personnel of the study (performance bias), blinding of outcome assessment (detection bias), incomplete outcome data (attrition bias), selective reporting of outcomes (reporting bias), and other bias. These features were evaluated as low risk of bias (+) when criteria were met and sufficiently described, high risk of bias (−) when criteria were not met, or unclear risk of bias (?) when information provided does not suffice for evaluation. Results of risk of bias assessments are reported in **Tables 2** and **4**.

**Table 2:** Risk of bias of included studies listed in **Table 1**.

	Random sequence generation (selection bias)	Allocation concealment (selection bias)	Blinding of participants and personnel (performance bias)	Blinding of outcome assessment (detection bias)	Incomplete outcome data (attrition bias)	Selective reporting (reporting bias)	Other bias
Gupta et al. (23)	+	?	?	+	+	−	?
Pattamatta et al. (24)	+	?	−	?	−	?	?

## Results

### Studies Reporting Hees for Placebo Interventions

**Figure 1:**
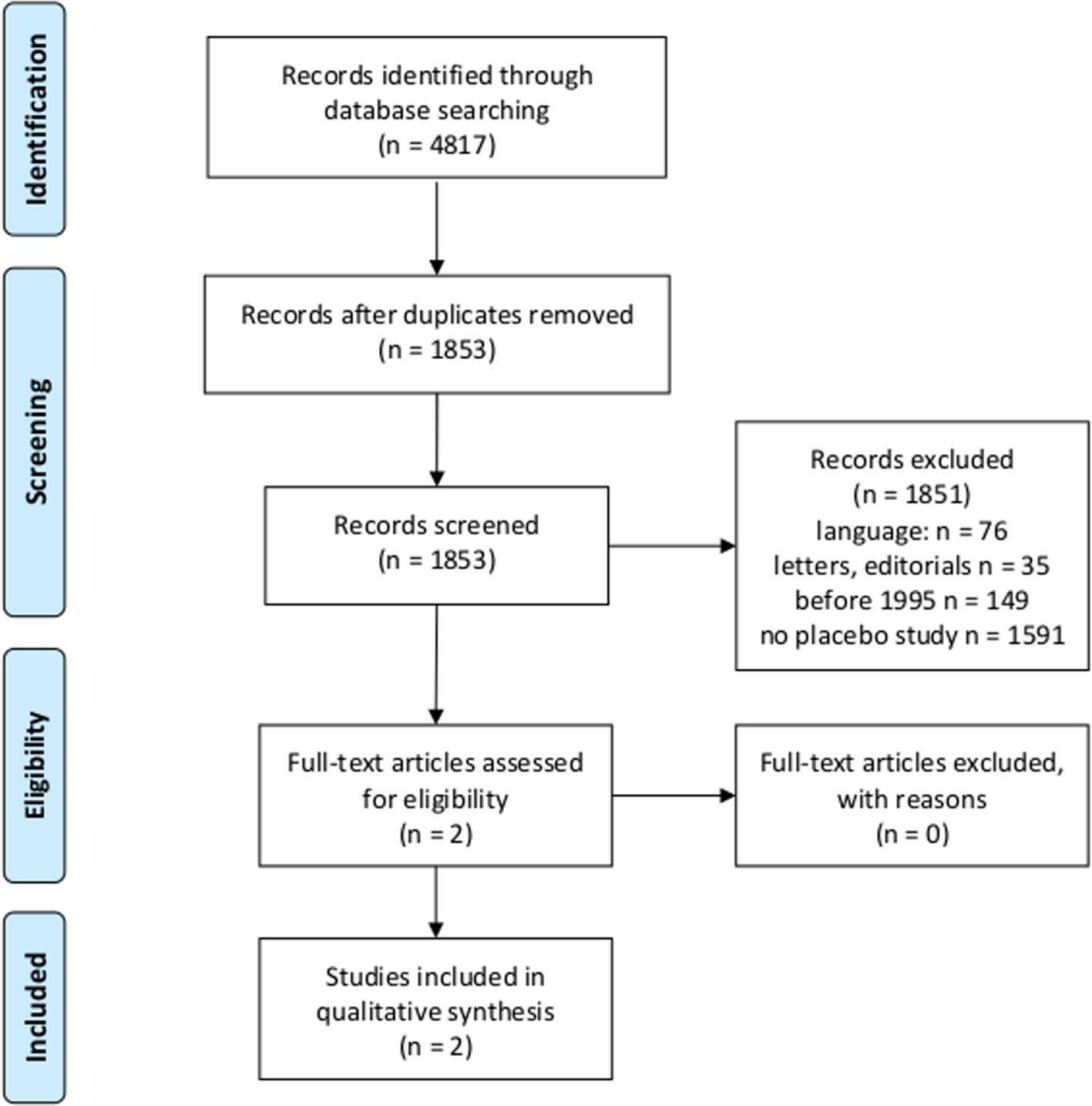
Flowchart according to the Preferred Reporting Items for Systematic Reviews and Meta-Analyses (PRISMA) statement (18, 19) for first research question.

After eligibility criteria were first screened, titles and abstracts of 1,593 studies were screened for the question whether they report a HEE of a placebo intervention in patients with any disorder or disease (**Figure 1**).

Two articles were identified that met the criteria (**Table 1**), and risk of bias was assessed (**Table 2**).

Gupta et al. (23) describe their intervention of using a flavored anesthetic mask as a placebo intervention by themselves and compared it with a non-flavored mask for children who undergo surgery. They report higher total costs for flavored compared with non-flavored masks (56.45 Indian rupee versus 54 Indian rupee) but did not relate it to effects of the masks. Pattamatta et al. (24) investigated if chewing a gum compared with a placebo dermal patch 3 h before and after colorectal surgery decreases complications such as postoperative ileus (PI) and anastomotic leakage (AL). Chewing a gum was considered a placebo intervention, as authors of this re-analysis of data did not provide any information about active mechanisms, and authors of the original article reported that the underlying mechanisms are still elusive (25). Costs for ward stay were lower in the gum chewing group, compared with the control group, but overall costs of treatment were not different. Calculation of ICERs for PI and AL (INR −2,414 and INR −8,450, respectively) showed superiority for the gum chewing group. Health-related quality of life was assessed but not used to calculate QALYs, as the author considered it inappropriate because of varying time points for the postoperative assessment.

### Risk of Bias in Studies Reporting Hees for Placebo Interventions

Both studies (23, 24) report randomization of patients, but it is unclear if a selection and other biases could have occurred due to insufficient description. Gupta et al. (23) report that patients were blinded to the condition, but it must be assumed that they realized their group assignment when they smelled the flavor of the mask. In the study by Pattamatta et al. (24), patients were not blinded to the condition as they differed in their form of application (chewing gum versus dermal patch) (**Table 2**).

### Studies Using Placebo Interventions and Outcomes Eligible for Hees

**Figure 2:**
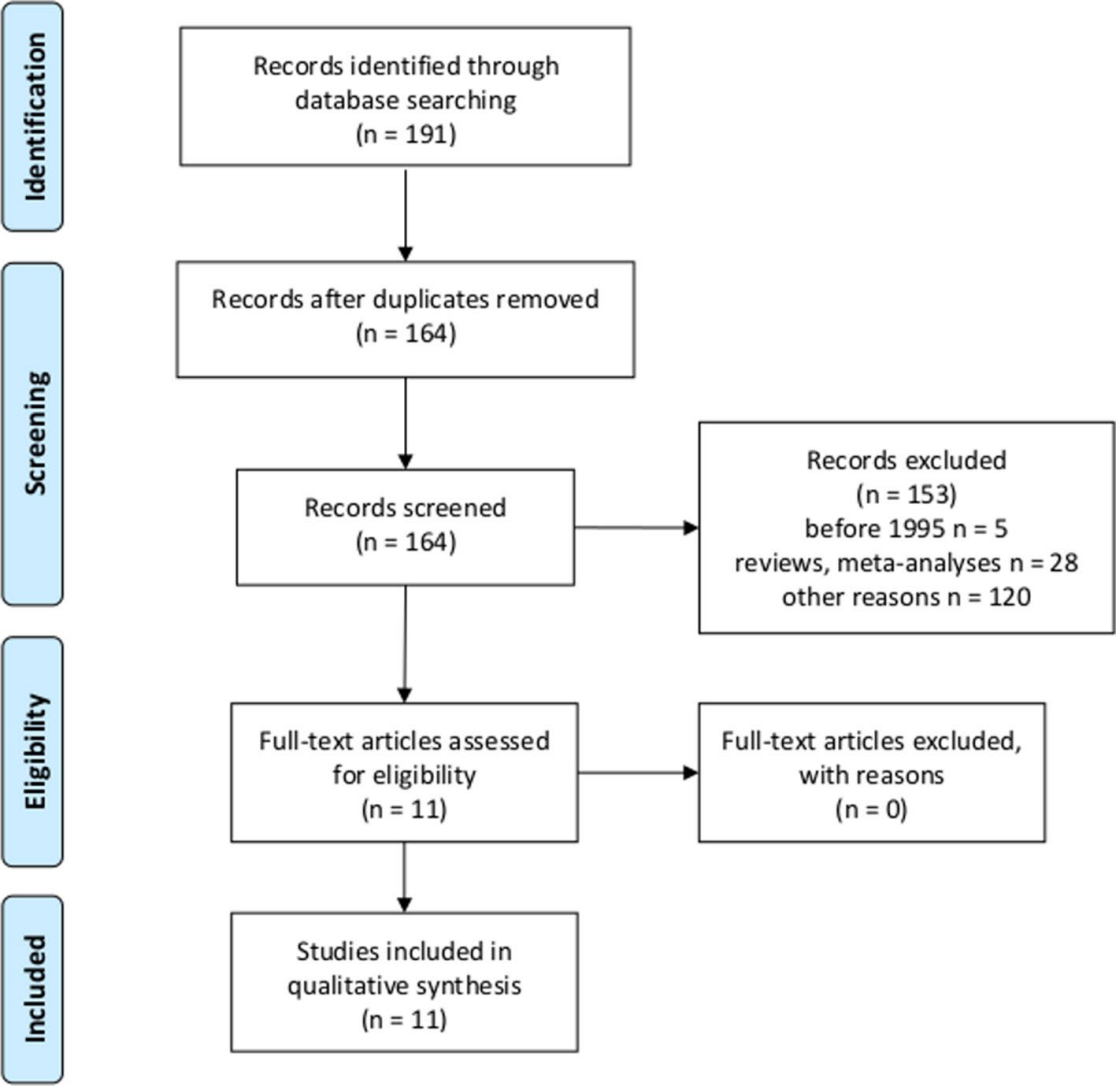
Flowchart according to the Preferred Reporting Items for Systematic Reviews and Meta-Analyses (PRISMA) statement (18, 19) for second research question.

Literature research using the JIPS database yielded 11 studies investigating intentional placebo interventions or mechanisms in comparison with control groups (**Figure 2**), and which assessed outcomes eligible for HEEs such as quality of life, morbidity, and mortality (**Table 3**). In six studies, patients received placebo pills in open-label conditions; that is, they knew that they received placebo pills only, in combination with an explanation on how they work and improve symptoms to increase treatment expectations (8, 9, 11, 26–28). In three studies, enhanced and particularly empathic patient–physician relationships were applied to enhance expectations of patients (15, 29, 30). Two studies used psychological interventions developed to optimize expectations concerning treatment outcomes (31, 32). Placebo interventions and mechanisms were applied to adult patients suffering from various diseases and disorders: with gastrointestinal disorders (8, 29, 30), respiratory or allergic diseases (11, 27, 31), cancer-related fatigue (26, 28), common cold (15), chronic low back pain (9), and heart surgery (32). Outcome measures eligible for HEEs were patient-reported general or disease-specific quality of life questionnaires such as different versions of the Short Form Health survey (SF-8, SF-12, and SF-36), specific questionnaires for IBS, gastroesophageal reflux disease (GERD), asthma, fatigue, and a disability questionnaire.

**Table 3:** Studies employing placebo interventions and reporting of outcome measures suitable for HEEs.

Study	Condition	Placebo intervention	Control group	No. of patients (male:female)	Age (years; M ± SD)	Outcome measures for HEEs
Kaptchuk et al. (29)	Irritable bowel syndrome (IBS)	(1) Placebo acupuncture alone (2) Placebo acupuncture plus augmented patient–practitioner relationship	Waiting list	262 (63:199)	39 ± 14	Global improvement scale (range 1–7), adequate relief of symptoms, IBS symptom severity scale, IBS quality of life
Rakel et al. (15)	Common cold	Enhanced physician visit	(1) Standard physician visit (2) No physician visit	350 (126:224)	36 ± 15	Cold severity score, duration of cold, quality of life (SF-8), feeling thermometer of the EuroQoL
Kaptchuk et al. (8)	IBS	Open-label placebo pills	No-treatment control	80 (24:56)	47 ± 18	IBS Global Improvement Scale, IBS Symptom severity scale, IBs Adequate relief, IBS QoL
Clerisme-Beaty et al. (31)	Suboptimally controlled asthma	Educational program to enhance expectations in placebo and drug group (enhanced/placebo, enhanced/montelukast)	Standard educational program in placebo and drug group (neutral/placebo, neutral/montelukast)	99 (28:71)	35 ± 15	Medication adherence, outcome expectancy, asthma outcomes (PEF, FEV1, ACQ), asthma QoL
Dosset et al. (30)	Gastroesophageal reflux disease (GERD)	Expanded empathic visit in placebo and homeopathic group	Standard empathic visit in placebo and homeopathic group	24 (8:16)	58 ± 11	Diary for GERD symptom severity, GERD health-related QoL, Gastrointestinal Symptom Rating Scale
Carvalho et al. (9)	Chronic low back pain	Open-label placebo pills	Treatment as usual (waitlist)	83 (24:59)	44 ± 13	Pain and bothersomeness on numeric rating scales, Roland-Morris Disability Questionnaire,
Schaefer et al. (11)	Allergic rhinitis	Open-label placebo pills	No treatment	25 (4:21)	26 ± 10	Self-developed allergic symptoms scale, QoL (SF-12)
Rief et al. (32)	Coronary artery bypass graft (CABG) surgery	Psychological intervention for expectation optimization	(1) Psychological intervention with emotional support (2) Standard medical care	115 (98:17)	66 ± 8	Pain Disability Index, QoL (SF-36), International Physical Activity Questionnaire (IPAQ), Hospital Anxiety and Depression Scale (HADS), Cardiac Anxiety Questionnaire
Hoenemeyer et al. (26)	Cancer-related fatigue	Open-label placebo pills	Treatment as usual (waitlist)	74 (19:55)	57 ± 12	Fatigue Symptom Inventory (FSI-14), Multidimensional Fatigue Symptom Inventory Short Form (MFSI-SF30) (measures QoL)
Schaefer et al. (27)	Allergic rhinitis	Open-label placebo pills with detailed information on placebos or no information	No pills with detailed information on placebos or no information	46 (9:37)	25 ± 7	Self-developed allergic symptoms scale, QoL (SF-36)
Zhou et al. (28)	Cancer-related fatigue	Open-label placebo pills	No treatment	40 (3:37)	47 ± 12	Functional Assessment of Chronic Illness Therapy-Fatigue (FACIT-F), QoL (SF-12), Profile of Mood States-Short Form (POMS-SF), Godin Leisure Time Exercise Questionnaire (GLTEQ)

HEEs, health economic evaluations; ICER, incremental cost-effectiveness ratio.

The MEDLINE research revealed *N* = 853 articles of which 472 were randomized controlled trials, 269 were no original studies (e.g., reviews, meta-analyses, and letters), 93 were other kind of studies (e.g., *post hoc* analyses of placebo arms of RCTs without control condition for other unspecific effects, or patients were not randomized to groups), and 14 studies did not involve patients. We identified five articles meeting our criteria, which have also been found in the JIPS database (8, 9, 27–29).

### Risk of Bias in Studies Using Placebo Interventions and Outcomes Eligible for Hees

Risk of bias according to the *Cochrane Handbook* is reported in **Table 4**. Most of the studies report adequate randomization, allocation concealment, and blinding of outcome assessment. Data were incomplete or insufficiently described in five studies, whereas selective reporting of results is assumed to occur only seldom. In 10 out of 11 studies, particularly patients and also practitioners were not blinded to the assigned condition.

**Table 4:** Risk of bias of identified studies listed in **Table 3**.

	Random sequence generation (selection bias)	Allocation concealment (selection bias)	Blinding of participants and personnel (performance bias)	Blinding of outcome assessment (detection bias)	Incomplete outcome data (attrition bias)	Selective reporting (reporting bias)	Other bias
Kaptchuk et al. (29)	+	+	−	+	+	+	−
Rakel et al. (15)	+	+	−	−	−	−	−
Kaptchuk et al. (8)	+	+	−	+	?	+	?
Clerisme-Beaty et al. (31)	?	+	?	?	?	?	?
Dossett et al. (30)	+	+	−	+	+	+	+
Carvalho et al. (9)	+	+	−	+	?	+	?
Schaefer et al. (11)	?	?	−	?	+	+	?
Rief et al. (32)	+	+	−	+	+	+	+
Hoenemeyer et al. (26)	+	+	−	+	+	+	?
Schaefer et al. (27)	+	+	−	+	?	+	?
Zhou et al. (28)	+	+	−	+	+	+	+

## Discussion

To provide a comprehensive overview of the current state of analyzed and potential HEEs of placebo effects, we performed two systematic reviews of the literature. The first searched for reported HEEs of placebo effects in studies involving patients with any disease or disorder who were treated with an intentional placebo intervention. We found two articles only matching these criteria, of which one was selected with some uncertainty as authors suspected an underlying active mechanism (cephalic vagal activation), and the control group was a placebo intervention, too (24). The latter could control for unspecific effects in both groups, but placebo effects of equal size could occur resulting in equal overall effects in both groups. However, they found that gum chewing was more effective than placebo dermal patch to reduce postoperative complications, and gum chewing had a better cost–benefit balance calculated as ICER. The other study (23) reported higher total costs for the placebo intervention compared with the control group, due to the fact that the control group was treatment as usual (unflavored anesthetic mask) compared with an intervention with additional preparations. Therefore, they chose a cost–cost analysis, calculating and comparing the costs of both alternatives only, but did not relate costs to effectiveness of treatment. Calculating ICER could have been more beneficial for the placebo intervention, as effects on anxiety behavior and compliance were better than in the control group. It should be mentioned that costs of placebo arms of RCTs were occasionally calculated and reported in articles found but were not considered in this review, as they serve only as a control group for a mixture of placebo effects and unspecific effects that are not meant to be used as intentional treatment. In summary, we found only two studies reporting HEEs for placebo interventions with medium to high risks of biases and limited analyses of costs and cost–benefit balances, which do not significantly contribute to knowledge about HEEs of placebo interventions.

Due to the minor result of this systematic review, despite a broad search strategy, we decided to perform a second literature research to answer the question if, at least, there are studies with patients that have investigated intentional placebo interventions and assessed outcomes that could be eligible for HEEs. This second search yielded 11 studies, which reported measures of quality of life (8, 9, 11, 15, 26–32), allowing to calculate ICURs or quality-adjusted life years (QALY) for placebo interventions when costs of treatments were known. These studies report a variety of placebo interventions such as open-label placebo pills, placebo acupuncture, educational programs to enhance expectations about the treatment, and expanded empathic visits, in different kinds of patients and disorders. HEEs could be calculated when costs of the applied placebo and control interventions are known and could then be compared with costs and effectiveness of standard treatments. For example, when all costs of an open-label application of placebo pills including pills, other materials, and working hours of physicians for the treatment of chronic low back pain (9) were known, they could be compared with total costs of standard treatments such as with analgesics. To calculate ICER, the effects of both treatments, such as an increase in quality of life or a decrease of symptoms, are compared in relation to their costs. Furthermore, the occurrence of side effects and the related costs of their treatment could be taken into account in further HEEs. However, the authors of the placebo studies did not report costs of interventions, as this was not the aim of their studies and articles. Risks for biases vary between low to medium among most studies, but all of them report that patients, and in some cases physicians, were not blinded to the condition. According to the *Cochrane Handbook* and risk of bias tool (22), this is deemed a performance bias, but the tool is designed to evaluate RCTs in which the placebo group is used to control for placebo responses, including the placebo effect *per se* as well as (other) unspecific effects such as regression to the mean and natural course of symptoms. In contrast, placebo interventions aim to intentionally utilize the placebo effect by increasing patients’ expectations. Blinding patients for their expectations being manipulated is very difficult to achieve and might be unethical, although not blinding patients could lead to better external validity than could blinded RCTs, as patients are not blinded to their treatment in daily routine.

## Limitations

Some limitations of our systematic literature reviews should be mentioned. In the first review, titles and abstracts were screened carefully for any hints that an intentional placebo intervention was applied. However, we cannot exclude that ineffective interventions were applied that could have been considered as placebo interventions. We relied on the assumption that authors who are aware of applying a placebo intervention use the words “placebo” or “placebos” in the title, abstract, or keywords of their articles. To double-check for additional articles that does not comprise “placebo” but used placebo mechanisms, we explored to search for “expectation OR expectancy” and “conditioning” in combination of the words listed in **Supplement 2**. These searches yielded too many inappropriate results; and we, therefore, did not implement them in our literature research. For the second review, we first screened the JIPS database consisting of pre-selected articles about placebo effects and double-checked the results by searching for “placebo effect,” “placebo response,” and “placebo treatment” in combination with pre-defined search terms for HEEs in MEDLINE/PubMed for any additional results. We thus restricted the search to articles explicitly referring to these effects and did not perform a broadly based search for “placebo” only. This MEDLINE research yielded five studies only (8, 9, 27–29), which were also found in the JIPS database. These five studies investigated placebo treatments using placebo pills or acupuncture, whereas the additional six studies harnessing psychological interventions were not detected with the search terms “placebo effect,” “placebo response,” or “placebo treatment.” Finally, CEAs could also be performed with other patient-reported outcomes (PROs) than those related to quality of life, for example, changes in any symptoms, or with biological parameters such as changes in inflammatory markers or heart rate variability. We restricted our search for measures of quality of life because they are most commonly used and recommended for HEEs and allow for comparisons between different kinds of treatments.

## Conclusions

The state of knowledge about HEEs of placebo interventions is scarce. To gain more visibility and acceptability for placebo interventions, we recommend that (1) future studies applying placebo interventions to patients should measure outcomes usable for HEEs, such as quality of life, morbidity or mortality (where appropriate), and costs of interventions, and (2) HEEs should be performed for existing studies that applied placebo interventions.

## Author Contributions

JH and KW contributed to the initial research questions for this systematic review and the search strategy and performed the literature research, screening of articles, data extraction, and quality assessment, and wrote the first draft of the manuscript. KM, TH, and TL contributed to the interpretation of results of literature research. All authors contributed to manuscript revision and read and approved the submitted version.

## Conflict of Interest Statement

The authors declare that the research was conducted in the absence of any commercial or financial relationships that could be construed as a potential conflict of interest.
