# Supplementary material for: Placebo Economics: A Systematic Review About the Economic Potential of Utilizing the Placebo Effect
Source: Front Psychiatry. 2019 Sep 12;10:653. doi: 10.3389/fpsyt.2019.00653 (PMC6751772; doi:10.3389/fpsyt.2019.00653)
Supplement: Supplementary file 1 [file DataSheet_1.docx]

Supplementary Material

Placebo economics: a systematic review about
the economic potential of utilizing the placebo effect

Jens Hamberger, Karin Meissner, Thilo Hinterberger, Thomas Loew, Katja Weimer^*^

*** Correspondence:** katja.weimer@uni-ulm.de

**Supplement 1**

PRISMA 2009 Checklist, according to (18).

| **Section/topic** | **#** | **Checklist item** | **Reported on page #** |
| --- | --- | --- | --- |
| **TITLE** | | |  |
| Title | 1 | Identify the report as a systematic review, meta-analysis, or both. | 1 |
| **ABSTRACT** | | |  |
| Structured summary | 2 | Provide a structured summary including, as applicable: background; objectives; data sources; study eligibility criteria, participants, and interventions; study appraisal and synthesis methods; results; limitations; conclusions and implications of key findings; systematic review registration number. | 1 |
| **INTRODUCTION** | | |  |
| Rationale | 3 | Describe the rationale for the review in the context of what is already known. | 2 f. |
| Objectives | 4 | Provide an explicit statement of questions being addressed with reference to participants, interventions, comparisons, outcomes, and study design (PICOS). | 3 |
| **METHODS** | | |  |
| Protocol and registration | 5 | Indicate if a review protocol exists, if and where it can be accessed (e.g., Web address), and, if available, provide registration information including registration number. | 3 |
| Eligibility criteria | 6 | Specify study characteristics (e.g., PICOS, length of follow-up) and report characteristics (e.g., years considered, language, publication status) used as criteria for eligibility, giving rationale. | 3 |
| Information sources | 7 | Describe all information sources (e.g., databases with dates of coverage, contact with study authors to identify additional studies) in the search and date last searched. | 3 |
| Search | 8 | Present full electronic search strategy for at least one database, including any limits used, such that it could be repeated. | 3 and supplement |
| Study selection | 9 | State the process for selecting studies (i.e., screening, eligibility, included in systematic review, and, if applicable, included in the meta-analysis). | 3 and figures 1 and 2 |
| Data collection process | 10 | Describe method of data extraction from reports (e.g., piloted forms, independently, in duplicate) and any processes for obtaining and confirming data from investigators. | 3 f. |
| Data items | 11 | List and define all variables for which data were sought (e.g., PICOS, funding sources) and any assumptions and simplifications made. | 3 f. |
| Risk of bias in individual studies | 12 | Describe methods used for assessing risk of bias of individual studies (including specification of whether this was done at the study or outcome level), and how this information is to be used in any data synthesis. | 4 |
| Summary measures | 13 | State the principal summary measures (e.g., risk ratio, difference in means). | 3 |
| Synthesis of results | 14 | Describe the methods of handling data and combining results of studies, if done, including measures of consistency (e.g., I^2^) for each meta-analysis. | 3 |

**Supplement 2**

MeSH terms used for the systematic review according to the recommendations of Droste and Dintsios (2011) (21), and number of articles found [in November 2018]. All listed MeSH terms were combined with “placebo”, and searched in MEDLINE/PubMed, e.g. “placebo” AND “cost allocation”[MeSH Terms].

| **MeSH Term** | **N** |
| --- | --- |
| Costs and Cost Analysis | 1682 |
| - Cost Allocation | 0 |
| - Cost - Benefit Analysis | 1218 |
| - Cost Control | 83 |
| - Cost Savings | 64 |
| - Cost of Illness | 132 |
| - Cost Sharing | 2 |
| - - Deductibles and Coinsurance | 1 |
| - - Medical Savings Accounts | 0 |
| - Health Care Costs | 555 |
| - - Direct Service Costs | 6 |
| - - Drug Costs | 297 |
| - - Employer Health Costs | 3 |
| - - Hospital Costs | 57 |
| - Health Expenditures | 19 |
| - - Capital Expenditures | 0 |
| Economic Competition | 1 |
| Economics, Dental | 0 |
| - Fees, Dental | 0 |
| Economics, Hospital | 66 |
| - Hospital Charges | 9 |
| - Hospital Costs | 57 |
| Economics, Medical | 8 |
| - Fees, Medical | 4 |
| Economics, Nursing | 0 |
| Economics, Pharmaceutical | 90 |
| Fees and Charges | 27 |
| - Capitation Fee | 0 |
| - Fee-for-Service Plans | 3 |
| - Fees, Pharmaceutical | 9 |
| - - Prescription Fees | 2 |
| - Rate Setting and Review | 2 |
| Financial Management | 34 |
| - Accounting | 1 |
| - Budgets | 5 |
| - Contract Services | 0 |
| - Financial Management, Hospital | 0 |
| - Marketing of Health Services | 10 |
| Financing, Organized | 145 |
| - Insurance | 104 |
| - - Insurance Benefits | 1 |
| - - Insurance, Health | 86 |
| - - - Insurance, Long-Term Care | 0 |
| - - - Insurance, Medigap | 0 |
| - - - Insurance, Psychiatric | 0 |
| - Insurance, Health, Reimbursement | 34 |
| - Single-Payer System | 0 |
| - Medical Savings Accounts | 0 |

**Supplement 3**

Search term used for the second literature search in MEDLINE:

(„placebo effect“ OR „placebo response“ OR "placebo treatment") AND (“quality of life”[Title/Abstract] OR “QoL”[Title/Abstract] OR “disability”[Title/Abstract] OR “SF-36”[Title/Abstract] OR “SF36”[Title/Abstract] OR “SF-12”[Title/Abstract] OR “SF12”[Title/Abstract] OR “EQ-5D”[Title/Abstract] OR “EQ5D”[Title/Abstract] OR “morbidity”[Title/Abstract] OR “mortality”[Title/Abstract] OR “quality-adjusted life years”[Title/Abstract] OR “QALY”[Title/Abstract] OR “disability-adjusted life years”[Title/Abstract] OR “DALY”[Title/Abstract]) AND (“Humans”[Mesh]) AND (“1995/01/01”[Date - Publication] : “2019/03/08”[ Date - Publication]) AND (English[Language] OR German[Language])
